# Determinants of Hospital Length of Stay in Herpes Zoster Patients: A Four‐Year Retrospective Study

**DOI:** 10.1002/hsr2.71427

**Published:** 2025-10-28

**Authors:** Maryam Sadat Sadati, Yasamin Dehghan, Alireza Valizadeh Samakoush, Zahra Marzbannia, Fatemeh Hassannia, Mozhdeh Sepaskhah

**Affiliations:** ^1^ Department of Dermatology, School of Medicine Shiraz University of Medical Sciences Shiraz Fars Iran; ^2^ Molecular Dermatology Research Center, School of Medicine Shiraz University of Medical Sciences Shiraz Fars Iran; ^3^ Student Research Committee Shiraz University of Medical Sciences Shiraz Fars Iran; ^4^ Department of Pathology, School of Medicine Shiraz University of Medical Sciences Shiraz Fars Iran

**Keywords:** Herpes zoster, hospitalization, length of stay, risk factor, Varicella‐zoster virus (VZV)

## Abstract

**Background and Aims:**

The Varicella‐zoster virus (VZV) primarily causes chickenpox and can later reactivate as herpes zoster (HZ) from latent VZV in sensory ganglia. HZ generally presents with unilateral dermatomal pain and vesicular skin eruptions. Immunosuppression, advanced age, and underlying comorbidities increase the risk of severity, hospitalization, and longer length of stay (LOS), adding a substantial burden to the healthcare system. In this study, we examined factors influencing LOS in hospitalized HZ patients.

**Methods:**

We aimed to identify factors contributing to prolonged LOS in hospitalized patients with confirmed HZ at a tertiary referral center in southwestern Iran, from March 2020 to September 2024. We performed statistical analyses to explore the relationships between demographics, clinical features, comorbidities, medications, and laboratory findings (ESR, CRP) with LOS.

**Results:**

Of the 109 hospitalized HZ patients (50.5% female), the mean LOS was 6.1 ± 5.2 days. LOS showed significant correlations with elevated ESR (*r* = 0.304, *p* = 0.006), severe complications like encephalitis and Ramsay Hunt Syndrome (*p* < 0.001), and pre‐existing neurological and renal conditions (*p* < 0.05). No links were observed with age, CRP levels, gender, or immunosuppressant drug use.

**Conclusion:**

Severe neurological complications such as encephalitis and Ramsay Hunt syndrome, along with specific comorbidities and high ESR, notably extend hospital stays for HZ patients. These findings highlight the burden of severe HZ and identify key factors affecting LOS. By quantifying these elements, we provide practical insights to optimize inpatient care and emphasize the importance of prevention in high‐risk populations.

## 1 Introduction

Varicella‐zoster virus (VZV) is a neurotropic alpha‐herpesvirus responsible for causing chickenpox as a primary infection, typically acquired in childhood, and herpes zoster (HZ), which may occur later in life due to reactivation of latent VZV within the dorsal root sensory ganglia or cranial sensory nerves [1, 2]. The lifetime risk of developing HZ ranges between 25% and 30% in the general population, increasing significantly with age, particularly in patients aged 50 and older [3, 4].

The global incidence of HZ also remains consistently higher among older adults, mainly due to demographic shifts such as population growth and aging [5]. This is a concerning issue as individuals aged over 70 face the highest risk of severe neurological complications like encephalitis and Ramsay Hunt syndrome, particularly those with underlying immunosuppression or comorbidities such as hematopoietic cell transplants, cancer, human immunodeficiency virus (HIV), solid organ transplants, autoimmune diseases, diabetes, chronic kidney disease (CKD), and cardiovascular disease [6, 7]. Fortunately, the burden of disease, measured by disability‐adjusted life years, has shown a promising decline in recent cohorts, due to improved disease prevention and management strategies such as vaccination, especially in high‐risk groups [5, 6].

Herpes zoster (HZ) typically presents with a painful vesicular eruption that follows a unilateral dermatomal distribution. Patients often report constitutional symptoms and prodromal pain at least 48 h before the appearance of cutaneous lesions. Subsequently, characteristic papulovesicular lesions develop within the affected dermatome and may last up to 2–4 weeks [8]. HZ is accompanied by numerous complications, ranging from localized cutaneous sequelae—such as postherpetic neuralgia, dyspigmentation, hypertrophic or keloidal scarring, and bacterial superinfection—to systemic and neurological manifestations. Severe complications include ophthalmic involvement, meningoencephalitis, cerebellar ataxia, Guillain‐Barré syndrome, stroke, and visceral involvement such as myocarditis and pneumonitis. Ramsay‐Hunt syndrome may also occur due to cranial nerve involvement [8, 9].

The diagnosis of HZ is primarily clinical and based on a physical exam and characteristic signs and symptoms. Although confirmatory tests like Tzanck smear, polymerase chain reaction, or direct fluorescent antibody assay can provide rapid diagnosis, they are usually reserved for atypical presentations [10, 11]. Initiation of antiviral therapy within 72 h of the development of lesions is strongly recommended to reduce symptom duration and mitigate potential complications [12]. Acyclovir, famciclovir, and valacyclovir are currently FDA‐approved drugs for herpes zoster treatment [12, 13].

While HZ generally follows a self‐limiting course in immunocompetent individuals, severely immunocompromised patients may develop extensive, multi‐dermatomal involvement, which can become disseminated and lead to serious disabilities [14]. Recent studies suggest that the risk of increased hospitalization rates and prolonged length of stay (LOS) in patients with HZ is strongly associated with immunosuppressive conditions, including HIV infection, rheumatoid arthritis, systemic lupus erythematosus, malignancies, and immunosuppressive therapy. Furthermore, the likelihood of hospitalization and prolonged LOS is markedly higher in individuals aged 50 years and older [15, 16]. Additional risk factors include diabetes mellitus (DM), chronic obstructive pulmonary disease, CKD, depression, genetics, and physical trauma [16, 17, 18].

Hospitalization due to HZ represents a significant economic burden and considerably impacts patients' quality of life. In this study, we aimed to evaluate the factors that affect the LOS in patients with HZ. We analyzed demographic characteristics (such as age and gender), dermatomal distribution, patients' medication history, underlying comorbidities, and laboratory findings in relation to the length of hospitalization and the development of secondary complications. The results of this study provide practical insights that can be applied in both treatment planning and healthcare management strategies.

## 2 Methods

### 2.1 Study Design and Population

We conducted a retrospective, cross‐sectional study on all patients hospitalized with a confirmed diagnosis of herpes zoster (HZ) in the dermatology ward of Shahid Faghihi Hospital, a tertiary referral center in southwestern Iran, affiliated with Shiraz University of Medical Sciences. The study covered 4 years from March 2020 to September 2024. Diagnosis of HZ was confirmed by a board‐certified dermatologist based on clinical criteria and hospital documentation. The target population included all patients diagnosed with HZ who were admitted to our dermatology center during this period. Patients with Incomplete or inaccessible medical records were excluded. Moreover, participants without a definitive diagnosis of herpes zoster and patients with duplicate records, such as those with identical names or hospital IDs, were excluded from the analysis. Additionally, Patients with a prior history of recombinant zoster vaccination (RZV) were excluded from this cohort. RZV is not part of the national immunization program in Iran, and it is not administered routinely. Therefore, vaccination status was not a confounding variable in our analysis.

### 2.2 Data Collection

Given the design and nature of the study, a census sampling method was applied. All eligible cases within the defined time frame were enrolled in our study. Data were collected from hospital records using a standardized form, which was approved by the dermatology review board of Shiraz University of Medical Sciences. Collected variables included:

- Demographics: age, sex
- Clinical manifestations: distribution of lesions, number of affected dermatomes, presence of systemic involvement
- Comorbidities: underlying diseases (e.g., diabetes mellitus, hypertension, malignancy, autoimmune conditions)
- Medication history: particularly concurrent use of immunosuppressive drugs
- Laboratory findings: erythrocyte sedimentation rate (ESR) and C‐reactive protein (CRP)
- Outcome: length of hospitalization

Data were reviewed and recorded by a trained dermatologist under the supervision of the principal investigator.

### 2.3 Statistical Analysis

Descriptive statistics summarized patient characteristics. Univariate analysis assessed relationships between identified variables and LOS as the dependent variable, among individuals with confirmed HZ. Further, associations between LOS and clinical variables were tested using the Chi‐square and Fisher′s exact tests, as appropriate. A *p*‐value of < 0.05 indicated statistical significance. All data were analyzed using SPSS software (version 27).

We additionally performed multivariable analyses to control for potential confounding. The primary model was an ordinary least squares (OLS) regression of log‐transformed LOS with robust (HC3) standard errors. Prespecified covariates included age, gender, number of dermatomes, immunosuppressant use, ESR, CRP, ophthalmic complication, severe complication (encephalitis/meningitis/Ramsay Hunt), CKD, and pre‐existing neurological disease. Comorbidities were extracted from medical history fields and harmonized into binary indicators using prespecified rules. As a sensitivity analysis, we fit a multivariable logistic regression with prolonged LOS (≥ 7 days) as the outcome using the same covariates. Model coefficients are reported as adjusted ratios (e^β) for OLS and odds ratios (OR) with 95% CIs for logistic regression.

### 2.4 Ethical Considerations

The Ethics Committee of Shiraz University of Medical Sciences approved this study (IR.SUMS.MED.REC.1403.320). Patients' data were anonymized, and confidentiality was strictly maintained throughout the study. Informed consent was waived due to the retrospective nature of the study and use of anonymized data.

## 3 Results

### 3.1 Patient Population and Baseline Characteristics

This retrospective study included 109 patients hospitalized for herpes zoster (HZ). The cohort had a nearly equal gender distribution (50.5% female) and a mean age of 56.9 ± 15.6 years. The overall mean LOS was 6.1 ± 5.2 days. None of the hospitalized patients had a prior history of RZV. Intravenous acyclovir was the most common treatment, administered to 76.1% of patients. Key demographic, treatment, and inflammatory marker data are summarized in Table 1.

**TABLE 1:** Demographic and baseline clinical characteristics of hospitalized HZ patients (*N* = 109).

Variable	*N* (%)	Mean ± SD
Age (years)	—	56.94 ± 15.64 (11 to 91)
Sex		
‐ Male	54 (49.5)	—
‐ Female	55 (50.5)	—
Length of stay (days)	—	6.1 ± 5.2
Inflammatory markers		
ESR (mm/hr)	—	29.8 ± 27.5
CRP (mg/L)	—	18.6 ± 22.7
Immunosuppressant use	40 (36.7)	—
Prednisolone administration	46 (42.2)	
Acyclovir administration		
Intravenous (IV)	83 (76.1)	—
Oral (PO)	20 (18.3)	—
None	5 (4.6)	—

### 3.2 Clinical Presentation and Comorbidities

The most frequent anatomical sites of involvement were the face and neck (31.2%) and the ophthalmic dermatome (27.5%). The majority of patients (77.1%) presented with a single affected dermatome. Ophthalmologic issues were the most common systemic complication, identified in 33.9% of the cohort (Table 2). A substantial proportion of patients had pre‐existing medical conditions, with hypertension being the most prevalent comorbidity at 38.5%, followed by diabetes mellitus at 24.8% (Table 3).

**TABLE 2:** Clinical presentation and systemic complications of HZ (*N* = 109).

Feature	Category	Frequency (%)
Affected dermatome	Face & Neck	34 (31.2)
	Ophthalmic (Eye)	30 (27.5)
	Trunk	11 (10.1)
	Abdomen	10 (9.2)
	Lower extremity	10 (9.2)
	Other	14 (12.8)
Number of dermatomes	1	84 (77.1)
	2	17 (15.6)
	≥ 3	8 (7.3)
Systemic complications	Ophthalmologic	37 (33.9)
	Ramsay Hunt syndrome	7 (6.4)
	Encephalitis	4 (3.7)
	Meningitis	3 (2.8)
	Other neurologic/systemic	2 (1.8)

**TABLE 3:** Frequency of underlying comorbidities in hospitalized HZ Patients (*N* = 109).

Comorbidity	Frequency (%)
Hypertension (HTN)	42 (38.5)
Diabetes mellitus (DM)	27 (24.8)
Cardiovascular disease (CAD)	17 (15.6)
Cancer	15 (13.8)
Hypothyroidism	12 (11.0)
Systemic lupus erythematosus (SLE)	11 (10.1)
Rheumatoid arthritis (RA)	10 (9.2)
History of chemotherapy	8 (7.3)
Pre‐existing neurological disease	7 (6.4)
Transplant recipient	6 (5.5)
Chronic kidney disease (CKD)	3 (2.8)
HIV	2 (1.8)

### 3.3 Factors Associated With Length of Hospital Stay

Univariate analysis was performed to identify factors associated with an increased LOS. A statistically significant positive correlation was found between LOS and ESR (*r* = 0.304, *p* = 0.006). Severe systemic complications were a considerable factor in prolonged hospitalization. Specifically, patients with encephalitis had the longest mean LOS (19.3 days), followed by those with Ramsay Hunt syndrome (14.6 days) and meningitis (9.0 days) (*p* < 0.001 for all). Specific underlying comorbidities, including pre‐existing neurological disease and CKD, were also associated with significantly longer hospitalizations (*p* < 0.05). In contrast, no significant association was found between LOS and age, CRP levels, gender, or a history of immunosuppressant or prednisolone use (*p* > 0.05). Table 4 details the statistical analysis of factors associated with LOS.

**TABLE 4:** Analysis of factors associated with mean length of hospital stay (LOS).

(A) Continuous variables		
Variable	Correlation (*r*)	*p* value
Age	0.174	0.070
ESR	0.304	0.006
CRP	0.121	0.284

(B) Categorical variables			
Factor	Category	Mean LOS (days)	*p* value
Gender	Male	6.67	> 0.05
	Female	5.44	
HZ Complication	Skin	5.00	> 0.05
	Ophthalmic	5.95	< 0.001
	Meningitis	9.33	< 0.001
	Encephalitis	19.30	< 0.001
	Ramsay Hunt syndrome	14.60	< 0.001
	Genitalia	—	‐ (omitted, constant)
	Neurologic (other)	—	‐ (omitted, constant)
Comorbidity	Neurological disease	9.29	< 0.05
	No neurological disease	5.82	
	Chronic kidney disease (CKD)	9.00	< 0.05
	No CKD	5.96	
	Chemotherapy	5.25	> 0.05
	No chemotherapy	6.11	
	Transplant	5.50	> 0.05
	No transplant	6.08	
	Cardiovascular disease (CAD)	4.24	> 0.05
	No CAD	6.38	
	Rheumatoid arthritis (RA)	6.20	> 0.05
	No RA	5.27	
	Systemic lupus erythematosus (SLE)	6.13	> 0.05
	No SLE	5.33	
	Diabetes mellitus (DM)	6.28	> 0.05
	No DM	5.50	
	Hypertension (HTN)	6.39	> 0.05
	No HTN	5.89	
	Cancer	7.00	> 0.05
	No cancer	5.89	
	Hypothyroidism	6.14	> 0.05
	No hypothyroidism	5.25	
	HIV	—	> 0.05 (limited data)
Dermatome involvement	Eye	6.13	< 0.001
	Face & Neck	6.50	< 0.001
	Trunk	5.18	0.165
	Abdomen	5.60	0.200
	Upper extremity	5.20	0.022
	Lower extremity	6.20	0.005
	General	11.33	—
	Back	3.60	0.200
	Genitalia	—	‐ (omitted, constant)
Immunosuppressant use	Positive	5.83	> 0.05
	Negative	6.17	
Prednisolone administration	Positive	6.15	> 0.05
	Negative	5.96	
Acyclovir administration	Intravenous (IV)	6.23	> 0.05
	Oral (PO)	4.65	
	None	9.20	

In the multivariable OLS model of log(LOS) (*n* ≈ 80 due to lab missingness), ESR exhibited a positive association with LOS (per 10 mm/hr increase; adjusted ratio ≈ 1.07, *p* ≈ 0.10). Severe complications (adjusted ratio ≈ 1.67–1.82), ophthalmic complication (≈1.35–1.39), and pre‐existing neurological disease (≈2.62–2.72) were also associated with longer adjusted LOS (all *p* ≈ 0.09–0.14). Age, gender, number of dermatomes, immunosuppressant use, CRP, and CKD were not significantly associated with LOS after adjustment. In the logistic model for prolonged LOS ≥ 7 days (*n* ≈ 78), neurological disease showed the largest effect (OR ≈ 9.3, 95% CI ~ 0.70–124; *p* ≈ 0.09), and female gender showed lower odds (OR ≈ 0.36; *p* ≈ 0.07). While precision was limited, the direction and magnitude of effects across models consistently implicated inflammatory burden and neuro‐ophthalmic complications as principal determinants of LOS (Table 5).

**TABLE 5:** Multivariable determinants of length of stay.

(A) OLS regression of log‐transformed LOS (*n* = 80). Results reported as adjusted ratios (e^β) with 95% CI and *p*‐values. Robust SEs used		
Variable	Adjusted ratio (95% CI)	*p*
Age	1.00 (0.99–1.07)	0.76
Gender	0.87 (0.61–1.26)	0.46
Dermatomes	1.17 (0.89–1.55)	0.25
Immunosuppressant	1.03 (0.64–1.65)	0.91
ESR	1.01 (1.00–1.04)	0.10
Severe complication	1.67 (0.86–3.25)	0.14
Ophthalmic complication	1.35 (0.93–1.97)	0.13
CKD	1.42 (0.38–5.30)	0.60
Neurological disease	2.72 (0.90–8.20)	0.12

(B) Logistic regression of prolonged LOS (≥ 7 days, *n* = 78). Results reported as adjusted odds ratios (OR) with 95% CI and *p*‐values.		
Variable	OR (95% CI)	*p*
Age	1.03 (0.99–1.07)	0.15
Gender	0.36 (0.12–1.09)	0.07
Dermatomes	1.06 (0.66–1.71)	0.80
Immunosuppressant	1.05 (0.26–4.25)	0.95
ESR	1.02 (0.99–1.04)	0.18
CRP	0.99 (0.96–1.02)	0.65
Severe complication	1.82 (0.36–9.09)	0.47
Ophthalmic complication	1.49 (0.43–5.19)	0.53
CKD	0.84 (0.03–23.3)	0.92
Neurological disease	9.31 (0.70–124.3)	0.09

## 4 Discussion

We designed and conducted this retrospective study to evaluate the factors that may contribute to the longer duration of hospitalization in patients with herpes zoster. Our study demonstrated that the primary drivers of prolonged hospitalization for HZ are the severity of clinical complications and specific patient comorbidities, rather than demographic factors. The presence of severe systemic complications like encephalitis (mean LOS: 19.3 days), Ramsay Hunt syndrome (mean LOS: 14.6 days), and meningitis (mean LOS: 9.0 days), or underlying conditions such as pre‐existing neurological disease (mean LOS: 9.3 days) and CKD (mean LOS: 9.0 days), were associated with significantly longer hospital stays. A statistically significant positive correlation was found between the LOS and ESR (*r* = 0.304, *p* = 0.006), suggesting that a higher inflammatory burden contributes to longer hospitalizations. However, this association did not extend to CRP, for which no significant correlation with LOS was found. Similarly, demographic factors did not show a significant link to LOS. While an observational difference in hospitalization duration was noted between men (mean, 6.7 days) and women (mean, 5.4 days), this difference was not statistically significant. These adjusted analyses reinforce that, beyond demographics and immunosuppression, inflammatory burden (ESR) and neuro‐ophthalmic complications account for most variability in LOS, even when controlling for confounders.

Consistent with previous research, our study identified severe neurological complications, such as encephalitis and Ramsay Hunt syndrome, as the primary drivers of prolonged hospitalization. For instance, large‐scale retrospective studies by Ishikawa et al. (2022) and Loubet et al. (2024) have independently confirmed that neurological involvement is a significant predictor of more extended hospital stays and increased morbidity [19, 20]. Furthermore, comprehensive reviews by Gilden et al. (2015) have affirmed that these complications often necessitate intensive care [21]. These complications require intensive interventions, such as IV acyclovir and corticosteroids, due to viral‐mediated neural inflammation [22]. Moreover, the protracted and often incomplete recovery trajectories associated with such neural injury contribute to more extended stays [23]. The prognosis for facial nerve function in Ramsay Hunt Syndrome, for instance, is highly time‐dependent; the significant drop in recovery rates from approximately 70%–75% with early treatment to as low as 30% with delayed intervention necessitates more extensive inpatient rehabilitation [24].

In addition, specific underlying comorbidities significantly increased the LOS in our study. The association with CKD is particularly notable, given that the first‐line treatment, acyclovir, is renally cleared [25]. In patients with impaired renal function, management is complicated by the need for meticulous dose adjustments and monitoring to prevent drug‐induced nephrotoxicity, thereby extending the period of inpatient care [26]. Similarly, patients with a pre‐existing neurological disease were found to have longer hospitalizations, which may be attributed to an increased vulnerability to more severe HZ‐related sequelae and the diagnostic challenge of distinguishing new deficits from underlying symptoms [27, 28]. This is consistent with prior evidence that a higher burden of comorbidity increases the risk of severe HZ complications, leading to prolonged hospital courses [28].

Unexpectedly, our analysis did not find a significant association between LOS and either advanced age or immunosuppression. This contrasts with previous reports where advanced age is a strong predictor of longer hospitalization, with some studies reporting a median LOS of 10 days for patients older than 74 years, a finding attributed to immunosenescence and a higher burden of comorbidities [1, 15, 19]. Likewise, immunosuppression is a well‐established risk factor for increased hospital admissions and prolonged LOS, as these patients have a higher risk of severe or disseminated disease that requires more intensive management, such as isolation and extended antiviral treatment [20, 29, 30]. The divergence of our findings from this established evidence may be attributable to several factors. One possible explanation for the lack of an age association is a potential masking effect; the presence of a severe complication, such as encephalitis, may be a powerful determinant of LOS that overshadows the independent contribution of age. Moreover, the mean age of our cohort (56.9 years) is younger than that in other studies that found a significant association. Regarding immunosuppression, the absence of an important finding in our cohort could potentially be attributed to the sample size of these patients (*n* = 40) being insufficient to detect a statistically significant difference. Additionally, standardized use of IV acyclovir in 76.1% of patients may have mitigated the impact of immunosuppression on LOS.

This study has limitations inherent to its design. As a single‐center, retrospective analysis, the findings may have limited generalizability, and the results depend on the accuracy and completeness of the existing medical records, introducing a risk of information bias from incomplete documentation. Additionally, selection bias may arise from including only hospitalized patients at a tertiary referral center, potentially excluding milder HZ cases and over‐representing severe ones. The relatively modest sample size, particularly for subgroups with severe complications like encephalitis (*n* = 4), may have limited our statistical power to detect significant associations for all variables. Variations in treatment protocols or unmeasured confounders, such as clinician preferences, may also have influenced LOS. The study also did not account for socioeconomic factors or potential disparities in healthcare access, which may influence patient outcomes and LOS. Globally, socioeconomic barriers like the high cost of modern vaccines can limit their uptake, particularly in developing nations, impacting the overall burden of VZV‐related disease [31]. While our study focused on clinical factors post‐hospitalization, future research in our region should investigate how these socioeconomic determinants affect the initial risk of severe HZ and the need for admission. Additionally, vaccination status (varicella/zoster) was not recorded in the data set and could not be evaluated as a modifiable risk factor; future work should capture and adjust for vaccination to quantify its impact on hospitalization and LOS.

Despite these limitations, a key strength of our study was the use of real‐world clinical data to identify the most critical drivers of resource utilization in hospitalized HZ patients. By analyzing a wide range of clinical variables, we were able to underscore that once a patient with HZ is hospitalized, Severe neurological complications and specific high‐risk comorbidities have a greater impact on LOS than demographic factors. These findings suggest that early risk stratification, through routine ESR testing and monitoring for neurological or renal complications, can guide tailored antiviral dosing and discharge planning, potentially reducing hospital resource strain. These insights suggest specific strategies for clinicians, such as implementing early intervention protocols for high‐ESR patients with neurological or renal comorbidities, and for policymakers, including targeted vaccination campaigns to reduce HZ incidence and hospitalization burden in high‐risk populations. Future prospective, multi‐center studies are warranted to validate these findings and to elucidate further the mechanisms by which these factors extend hospitalization.

It is crucial to frame these findings within the context of prevention through immunization, as vaccination remains the most effective long‐term strategy for reducing the burden of HZ [31]. Immunization strategies typically involve a two‐dose varicella vaccine schedule for children and a highly effective recombinant zoster vaccine for adults over 50 [31]. In many countries, these vaccines are offered actively and free of charge through national immunization programs to ensure high coverage [32]. The medium to long‐term impact of these campaigns on HZ incidence and hospitalization is significant. Mathematical models based on universal childhood varicella vaccination predict a substantial long‐term reduction in HZ cases and associated hospitalizations—by as much as 58%—as vaccinated cohorts age [33]. However, these models also suggest the possibility of a temporary increase in HZ incidence for several decades following the introduction of varicella vaccination, theoretically due to reduced opportunities for natural immunity boosting from circulating VZV [33]. Despite this transient risk, the clear long‐term benefit underscores that strengthening both pediatric varicella and adult zoster immunization campaigns is the most impactful public health approach to mitigating severe HZ and reducing the need for hospitalization.

## 5 Conclusion

Overall, the data provided in this cross‐sectional study suggest that severe neurological complications, particularly encephalitis and Ramsay Hunt Syndrome, along with specific underlying comorbidities, remain the major determinants of prolonged hospitalization in patients with herpes zoster. Furthermore, higher levels of ESR were significantly associated with increased LOS in this cohort (*p* < 0.05). These findings underscore the substantial clinical and economic burden of HZ, particularly among high‐risk populations. By quantifying key drivers of extended hospitalization, our results provide actionable evidence to optimize inpatient management protocols. Prioritization of HZ vaccination among high‐risk groups could also play a crucial role in mitigating severe complications and hospitalizations due to this condition. Future research should aim to overcome the limitations of hospitalization‐based data by employing prospective, population‐level studies that can capture the full spectrum of disease severity, including cases managed in outpatient settings.

## Author Contributions

Maryam Sadat Sadati is responsible for study design, statistical analysis, and revision of the manuscript. Yasamin Dehghan conducted statistical analysis, wrote the draft of the manuscript, and revision of the manuscript. Alireza Valizadeh Samakoush and Zahra Marzbannia are responsible for data gathering and interpretation. Fatemeh Hassannia wrote the draft of the manuscript. Mozhdeh Sepaskhah provided technical support. All authors have read and approved the final version of the manuscript.

## Ethics Statement

The ethical review committee of the Shiraz University of Medical Sciences approved the study.

## Conflicts of Interest

The authors declare no conflicts of interest.

## Transparency Statement

The lead author, Yasamin Dehghan, affirms that this manuscript is an honest, accurate, and transparent account of the study being reported; that no important aspects of the study have been omitted; and that any discrepancies from the study as planned (and, if relevant, registered) have been explained.

## Data Availability

The data that support the findings of this study are available from the corresponding author upon reasonable request. Dr. Yasamin Dehghan had full access to all of the data in this study and takes complete responsibility for the integrity of the data and the accuracy of the data analysis.
